# Severity assessment to guide empiric antibiotic therapy for cholangitis in children after Kasai portoenterostomy: a multicenter prospective randomized control trial in China

**DOI:** 10.1097/JS9.0000000000000682

**Published:** 2023-09-02

**Authors:** Pei Wang, Hong-yi Zhang, Jixin Yang, Tianqi Zhu, Xiaojuan Wu, Bin Yi, Xiaoyi Sun, Bin Wang, Tao Wang, Weibing Tang, Hua Xie, Jinfa Tou, Yijiang Han, Xiang Liu, Jianghua Zhan, Yuanmei Liu, Yingchao Li, Zhibao Lv, Li Lu, Baohong Zhao, Tingliang Fu, Dianming Wu, Jianxi Bai, Wanfu Li, Heying Yang, Guofeng Zhang, Hongxia Ren, Jiexiong Feng

**Affiliations:** a Department of Pediatric Surgery, Tongji Hospital, Tongji Medical College of Huazhong University of Science and Technology; Hubei Clinical Center of Hirschsprung Disease and Allied Disorders, Wuhan; b Department of General Surgery, Shenzhen Children’s Hospital, Guangdong; c Department of Neonatal Surgery, Children’s Hospital of Nanjing Medical University, Jiangsu; d Department of Neonatal Surgery, Zhejiang University School of Medicine Children’s Hospital, Zhejiang; e Department of Pediatric Surgery, Anhui Provincial Children’s Hospital, Anhui; f Department of Neonatal Surgery, Children’s Hospital of Shanxi, Shanxi; g Department of General Surgery, Tianjin Children’s Hospital, Tianjin; h Department of Pediatric General Thoracic and Urology Surgery, The Affiliated Hospital of Zunyi Medical University, Guizhou; i Department of Surgery, Second Hospital of Hebei Medical University, Hebei; j Department of General Surgery, Children’s Hospital of Shanghai, Shanghai Jiao Tong University, Shanghai; k Department of Pediatric Surgery, Affiliated Hospital of Binzhou Medical College, Shandong; l Department of General Surgery and Oncology, Fujian Maternity and Child Health Hospital, Affiliated Hospital of Fujian Medical University, Fujian; m Department of Pediatric Surgery, First Affiliated Hospital of Xinjiang Medical University, Urumqi; n Department of Pediatric Surgery, The First Affiliated Hospital of Zhengzhou University, Zhengzhou, People’s Republic of China

**Keywords:** biliary atresia, cholangitis, intravenous antibiotics, Kasai portoenterostomy

## Abstract

**Background:**

Cholangitis is common in patients with biliary atresia following Kasai portoenterostomy (KPE). The prompt use of empiric antibiotics is essential due to the lack of identified microorganisms. The authors aimed to validate a severity grading system to guide empiric antibiotic therapy in the management of post-KPE cholangitis.

**Materials and methods:**

This multicenter, prospective, randomized, open-label study recruited patients with post-KPE cholangitis and was conducted from January 2018 to December 2019. On admission, patients were categorized into mild, moderate, and severe cholangitis according to the severity grading system. Patients in the mild cholangitis group were randomized to receive cefoperazone sodium tazobactam sodium (CSTS) or meropenem (MEPM). Patients with severe cholangitis were randomized to treatment with MEPM or a combination of MEPM plus immunoglobulin (MEPM+IVIG). Patients with moderate cholangitis received MEPM.

**Results:**

The primary endpoint was duration of fever (DOF). Secondary outcomes included blood culture, length of hospital stay, incidence of recurrent cholangitis, jaundice clearance rate, and native liver survival (NLS). For mild cholangitis, DOF, and length of hospital stay were similar between those treated with CSTS or MEPM (all *P*>0.05). In addition, no significant difference in recurrence rate, jaundice clearance rate, and NLS was observed between patients treated with CSTS and MEPM at 1-month, 3-month, and 6-month follow-up. In patients with moderate cholangitis, the DOF was 36.00 (interquartile range: 24.00–48.00) h. In severe cholangitis, compared with MEPM, MEPM+IVIG decreased DOF and improved liver function by reducing alanine aminotransferase, aspartate aminotransferase, gamma-glutamyl transferase, and direct bilirubin at 1-month follow-up. However, recurrence rate, jaundice clearance rate, and NLS did not differ significantly between MEPM+IVIG and MEPM at 1-month, 3-month, and 6-month follow-up.

**Conclusions:**

In patients with post-KPE cholangitis, MEPM is not superior to CSTS for the treatment of mild cholangitis. However, MEPM+IVIG treatment was associated with better short-term clinical outcomes in patients with severe cholangitis.

## Introduction

Highlights

- The severity grading system was feasible and effective in guiding empiric antibiotic therapy for post-Kasai portoenterostomy cholangitis.
- For mild cholangitis, cefoperazone is the first-line antibiotic.
- Meropenem is the optimal antibiotic treatment for moderate cholangitis.
- Meropenem plus immunoglobulin treatment could be used for those with severe cholangitis.

Biliary atresia (BA) is a progressive fibroobliteration of the biliary tract that causes cholestatic jaundice in infants^1^. The incidence of BA ranges from 1: 8000 to 1: 18 000 live births^2^. Kasai portoenterostomy (KPE) is the first-line treatment to restore bile flow^3^. Cholangitis is the most common complication after KPE^4,5^. It occurs in 30–70% of BA patients within 6 months of surgery, with an overall incidence of 40–93%^4^. Frequent episodes of cholangitis are associated with a poor prognosis^6^.

As with many other bacterial infections, appropriate treatment is usually guided by culture results. Unfortunately, reported positive culture rates in post-KPE cholangitis ranged from 8.9 to 26.9%^1,4,7,8^. The use of empiric antibiotics is often mandatory in the acute presentation of cholangitis. Cefoperazone has been used as a first-line antibiotic^9,10^. However, the efficacy of cefoperazone has decreased over the years^10^. In many institutions, meropenem (MEPM) has been proven to be an effective antibiotic^7,11,12^. Recently, intravenous immunoglobulin (IVIG) has shown promising results as an effective adjunctive treatment for refractory post-KPE cholangitis^11,13^.

In this study, we propose criteria for grading the severity of post-KPE cholangitis. The severity of cholangitis is divided into three grades at admission: mild, moderate, and severe. The empirical antibiotic regimen was selected accordingly among cefoperazone, MEPM, or MEPM+IVIG. Clinical outcomes were analyzed to determine the feasibility and efficacy of severity-guided empiric antibiotic use in the management of post-KPE cholangitis.

## Materials and methods

### Study design

This is a multicenter, prospective, open-label, randomized, controlled trial. It was conducted in 14 hospitals in China.

### Participants

Patients with an episode of cholangitis was defined as the presence of unexplained fever (≥38°C) combined with at least one of the following findings: recurrent jaundice or acholic stools; direct bilirubin (DBIL) greater than or equal to 20 µmol/l; white blood cell count (WBC) greater than or equal to 10×10^9^/l or C-reactive protein (CRP) greater than or equal to 10 mg/l^13,14^. Jaundice clearance was defined as a total serum bilirubin level less than 20 μmol/l.

Inclusion criteria were: patients diagnosed with type III BA who underwent KPE. The diagnosis of type III BA is based on intraoperative cholangiography in combination with histologic features of liver biopsies; suffering from an episode of cholangitis; not receiving other antibiotics or probiotics prior to enrollment; informed consent and willingness of their parents to cooperate with treatment and follow-up; not allergic to the medication. In addition, if the parents withdrew consent for any reason, or if the patient developed serious complications, the study would be discontinued and the subsequent alternative clinical management would be offered.

We excluded participants with known immunodeficiency, known viral infection, an identified source of infection other than cholangitis, end-stage liver failure, hemolytic jaundice, known allergies, or other contraindications to the use of IVIG, cefoperazone sodium tazobactam sodium (CSTS) and MEPM, a history of cholangitis unresponsive to CSTS, unstable conditions and sepsis-related organ damage, other liver disease or severe comorbidities requiring surgical intervention^11^.

Indications for discharge from the hospital were: fever-free for 72 h and normal levels of WBC (<10×10^9^/l) and CRP (<10 mg/l)^7^. Informed consent was obtained from the guardians of the participants prior to enrollment.

### Study protocol

The trial was approved by the Ethics Committee and registered on Clinicaltrial.gov. It is reported in accordance with the Consolidated Standards of Reporting Trials (CONSORT)^15^.

### Randomization and masking

The TG18 (Tokyo Guidelines 2018) classification grades the severity of cholangitis and guides clinical decisions in individual patients based on inflammatory markers, including WBC and CRP^9,14,16^. However, the values of WBC and CRP for assessing the severity of cholangitis in children have not been established. The severity grading criteria were proposed based on our previous study, in which we retrospectively analyzed the clinical outcome of 113 BA patients who had one or more episodes of post-KPE cholangitis^13^. Patients with WBC less than or equal to 15×10^9^/l, and CRP less than or equal to 30 mg/l was the lower limit of the interquartile range (IQR) for the median of WBC and CRP in the 113 patients. Meanwhile, patients with WBC greater than or equal to 20×10^9^/l, and CRP greater than or equal to 70 mg/l was the upper limit of the IQR for the median of WBC and CRP in the 113 patients. In this study, we defined the patients with mild cholangitis as those with T less than or equal to 38.5°C, WBC less than or equal to 15×10^9^/l, and CRP less than or equal to 30 mg/l. Severe cholangitis fulfilled the following criteria T greater than or equal to 39°C, WBC greater than or equal to 20×10^9^/l, and CRP greater than or equal to 70 mg/l. Patients were classified as having moderate cholangitis if they did not meet the criteria for either mild or severe cholangitis.

Patients with mild cholangitis were randomized (1:1) to receive intravenous injection of CSTS or MEPM. Patients with severe cholangitis were randomized (1:1) to receive intravenous MEPM or a combination of intravenous MEPM and immunoglobulin (MEPM+IVIG). The randomization sequence was generated using a web-based application (Microsoft Office Excel 2013, Microsoft Corporation). The randomization schedule was designed before the first patient was enrolled and did not change throughout the study. Participants, site staff, and treating physicians were not masked to treatment allocation. The random assignment sequences were concealed in the airtight envelopes. When an infant qualified for enrollment, the appropriate airtight envelope with the unique random number was given to the parent. The investigator would then open the envelope and obtain the detailed intervention information. In addition, to better control for bias, the clinical reviewers and the statistician were all blind to the allocation sequence.

After randomization, blood cultures and antimicrobial susceptibility testing were obtained prior to empiric antibiotic treatment. Antibiotics would be adjusted under the following conditions: different effective antibiotics as determined by antimicrobial susceptibility testing; CSTS would be changed to MEPM with or without IVIG if there was no initial rapid reduction in WBC and CRP; clinical deterioration, including shock, and persistent or worsening abdominal pain. The study would be stopped under these circumstances.

### Interventions

Patients with mild cholangitis received either CSTS at 50 mg/kg, Q12h or MEPM at 20 mg/kg, Q8h. Patients with moderate cholangitis received intravenous MEPM at 20 mg/kg, Q8h. Patients with severe cholangitis received either intravenous MEPM at 20 mg/kg, Q8h, or MEPM+IVIG (IVIG, 400 mg/kg, Q24h, for 3 days).

Other post-Kasai treatments were the same in all groups, including intravenous ornidazole at 20 mg. mg/kg, Q24h, steroids (oral prednisone at 5 mg mg/kg, Q24h, for 5 days), ursodeoxycholic acid (oral UDCA at 10 mg mg/kg, twice daily), liver-protective drugs, and vitamin A, vitamin D, and vitamin K supplements^2,17^.

### Study outcomes

Demographic characteristics and clinical characteristics were assessed, including gender, age, body weight, age at KPE, age at the first episode of cholangitis, body temperature, duration of fever subsided to normal (DOF) and length of hospital stay (LOS). Laboratory assessments included WBC, CRP, alanine aminotransferase (ALT), aspartate aminotransferase (AST), DBIL, gamma-glutamyl transferase (γ-GGT), and bacterial culture before the intervention. Follow-up visits were scheduled at 1, 3, and 6 months after discharge. The study endpoint was defined as: completion of 6-month follow-up; liver transplantation; death.

The primary outcome was DOF. If patients had a normal temperature after antipyretic therapy on admission, DOF was recorded as the time from admission to fever resolution. Secondary outcomes included blood culture, LOS, incidence of recurrent cholangitis, jaundice clearance, and native liver survival (NLS) at 1, 3, and 6 months^7^.

### Statistical analysis

The sample size was calculated based on the primary endpoint of DOF. In patients with mild cholangitis, CSTS was assumed to be non-inferior to MEPM with a noninferiority margin of 5 days. Assuming that the difference between the groups is 7 days (SD, 18), the minimum number of patients required in each group is 29 (α=0.05, power=80%). Considering an expected dropout rate of 10%, a total of 66 patients (33 in each group) with mild cholangitis were required. For patients with severe cholangitis, we assumed that MEPM+IVIG was superior to MEPM. Assuming that the difference between groups is 15 days (SD, 18), the minimum number of patients required in each group would be 24 (α=0.05, power=80%). Considering an expected dropout rate of 10%, a total of 52 patients (26 in each group) with severe cholangitis were required.

All analyses were performed on an intention-to-treat basis. Continuous variables are presented as mean (SD) or median (IQR) and compared using the Student’s *t*-test or Wilcoxon rank sum test. Categorical variables are presented as *n* (%) and compared using the *χ* ^2^ test or Fisher’s exact test. ANOVA analyses were performed when more than two groups of continuous variables were compared. Post-hoc pairwise comparisons were performed using the SNK test. Two-sided *P* values less than 0.05 were considered statistically significant. Post-hoc subgroup analyses of the primary outcome based on severity of cholangitis were performed without adjustment for significance. No imputation methods were used to impute missing values of baseline variables. Our analysis was based on the intention-to-treat principle; participants were analyzed by randomized group and not by treatment received. All statistical analyses were performed using IBM SPSS Statistics version 26.0 and SAS version 9.4. A *P*-value<0.05 was considered statistically significant.

## Results

### Study population

From January 2018 to December 2019, 269 patients from 14 hospitals were screened for eligibility (CONSORT flowchart).

A total of 42 patients were excluded from the study. Of these, 18 refused to participate in the study. The nine patients did not meet the inclusion criteria. The 13 patients received antipyretics or antibiotics prior to hospitalization and two were diagnosed with type I BA. Within 3 days of starting initial empiric treatment, 16 patients switched to a different antibiotic and dropped out of the study: 11 switched from CSTS to MEPM, 3 to quinolones, IVIG was added to MEPM in 2 patients. The reasons for changing antibiotics were as follows: no initial rapid reduction in WBC and CRP (2, 12.5%), clinical deterioration (6, 37.5%), and antibacterial susceptibility testing (8, 50.0%).

The remaining 211 patients were enrolled in the study. Of the 211 eligible patients, 67 were considered to have mild cholangitis, 90 had moderate cholangitis and 54 had severe cholangitis. A flowchart of participant eligibility and recruitment is provided in the CONSORT flowchart.

Table 1 shows the demographic and baseline characteristics of participants with mild and severe cholangitis. In the mild group, patients were randomized 1:1 to receive intravenous CSTS or MEPM. In the severe group, patients were randomized to receive intravenous MEPM or MEPM combined with immunoglobulin (MEPM+IVIG).

**Table 1:** Baseline characteristics of the patients.

	Mild cholangitis			Severe cholangitis		
	CSTS *N*=34	MEPM *N*=33	*P*	MEPM+IVIG *N*=26	MEPM *N*=28	*P*
Sex, *N* (%)						
Male	13 (38.24)	14 (42.42)	0.69	8 (30.77)	11 (39.29)	0.51
Female	21 (61.76)	19 (57.58)		18 (69.23)	17 (60.71)	
Median age, months, median (IQR)	6.50 (4.00–12.00)	9.00 (6.00–15.00)	0.86	5.85 (4.00–7.00)	6.15 (4.40–8.90)	0.41
Age at KPE, days, median (IQR)	55.50 (46.00–71.00)	55.00 (49.00–64.00)	0.47	70.00 (66.00–77.00)	77.00 (66.50–84.50)	0.34
AFC, median (IQR)	4.09 (3.40–8.00)	6.20 (5.00–9.00)	0.91	3.60 (3.00–4.00)	3.95 (3.55–4.55)	0.05
T, °C, median (IQR)	37.85 (37.50–38.60)	37.90 (37.50–38.40)	0.64	39.00 (38.90–39.50)	39.05 (38.80–39.50)	0.92
WBC, ×10^9^/l, median (IQR)	13.35 (10.00–16.04)	12.90 (10.80–15.20)	0.52	21.38 (18.00–25.40)	20.51 (18.17–25.01)	0.64
CRP, mg/l, median (IQR)	21.48 (12.08–33.40)	20.40 (18.00–30.90)	0.51	87.60 (67.70–109.00)	101.80 (75.75–121.20)	0.48
ALT, U/l, median (IQR)	139.65 (84.00–212.00)	164.00 (141.00–188.00)	0.68	256.00 (175.00–356.00)	286.00 (152.00–383.50)	0.48
AST, U/l, median (IQR)	175.00 (123.00–199.00)	172.00 (141.00–198.00)	0.462	232.00 (151.00–294.00)	266.75 (139.00–388.50)	0.28
γ-GGT, U/l, median (IQR)	470.50 (295.00–568.00)	488.00 (414.00–564.00)	0.45	755.50 (476.00–870.00)	752.00 (590.50–887.50)	0.59
DBIL, mg/dl, median (IQR)	39.10 (29.95–49.10)	48.00 (38.90–55.60)	0.75	94.75 (79.00–121.20)	81.80 (71.65–98.05)	0.83

AFC, age at first cholangitis; ALT, alanine aminotransferase; AST, aspartate aminotransferase; CRP, C-reactive protein; CSTS, cefoperazone sodium tazobactam sodium; DBIL, direct bilirubin; IQR, interquartile range; IVIG, intravenous immunoglobulin; KPE, Kasai portoenterostomy; MEPM, Meropenem; T, temperature; WBC, white blood cell; γ-GT, gamma-glutamyl transferase.

Median age was compared using Mann–Whitney test; Age at operation and DBIL were compared using independent samples *t*-test.

Baseline characteristics were balanced between the two subgroups in each group. In the mild cholangitis group, the mean age at KPE was 55.50 (IQR: 46.00–71.00) days in the CSTS group and 55.00 (IQR: 49.00–64.00) days in the MEPM group (*P*=0.471) (Table 1). In the severe cholangitis group, the mean age at KPE was 77.00 (IQR: 66.50–84.50) days in the MEPM group and 70.00 (IQR: 66.00–77.00) days in the MEPM+IVIG group (*P*=0.342). Baseline levels of other parameters, including age, sex, admission body temperature, WBC, CRP, and serum biochemical indicators of liver function (ALT, AST, γ-GGT, and DBIL) were not significantly different between the two subgroups in each group.

### Primary outcomes

In many centers, MEPM is a standard treatment for patients with moderate to severe post-KPE cholangitis^18,19^. To evaluate the therapeutic effects of MEPM, we compared the outcomes of patients with mild (*n*=33), moderate (*n*=90), and severe cholangitis (*n*=28) (Table 2). Sex, median age, age at first cholangitis and cholangitis episodes were not significantly different between the three groups. However, the severe cholangitis group had significantly higher body temperature, WBC count, CRP, ALT, AST, γ-GGT, and DBIL (Table 2). We found that the DOF was 28.00 (IQR: 24.00–40.00) h, 36.00 (IQR, 24.00–48.00) h, and 68.00 (IQR, 60.00–86.00) h in the patients with mild to severe cholangitis, which was significantly different among the three groups (*P*<0.0001) (Table 3). In addition, patients with severe cholangitis had the longest DOF, followed by moderate and mild cholangitis (Table 3). The age at KPE was significantly different between patients with mild, moderate, and severe cholangitis (*P*<0.0001) (Table 2). Previous studies have shown that early KPE before 60 days of age is associated with a better clinical outcome^18,20^. Therefore, we compared the therapeutic effects of MEPM in infants with different ages at KPE. We found that in infants with either KPE age less than or equal to 60 days or KPE age greater than 60 days, patients with severe cholangitis had the longest DOF, followed by moderate and mild cholangitis (Table 3). These observations indicated that the DOF increased with the severity of cholangitis, suggesting that we should optimize the use of empirical antibiotics according to the severity of cholangitis.

**Table 2:** The baseline characteristic of patients treated with MEPM for mild, moderate, and severe cholangitis.

	Mild cholangitis *N*=33	Moderate cholangitis *N*=90	Severe cholangitis *N*=28	*P*
Sex, *N* (%)				00.84
Male	14 (42.42)	33 (36.67)	11 (39.29)	
Female	19 (57.58)	57 (63.33)	17 (60.71)	
Median age, months, median (IQR)	9.00 (6.00–15.00)	7.25 (4.90–13.00)	6.15 (4.40–8.90)	00.31
Age at KPE, days, median (IQR)	55.00 (49.00–64.00)	63.00 (49.00–75.00)	77.00 (66.50–84.50)	<0.0001
AFC, months, median (IQR)	6.20 (5.00–9.00)	4.09 (3.50–6.00)	3.95 (3.55–4.55)	0.13
Episodes of cholangitis, median (IQR)	2.00 (1.00–2.00)	2.00 (1.00–2.00)	2.00 (1.00–3.00)	0.29
T, °C, median (IQR)	37.90 (37.50–38.40)	38.50 (37.60–39.00)	39.05 (38.80–39.50)	<0.0001
WBC, ×10^9^/l, median (IQR)	12.90 (10.80–15.20)	16.04 (12.12–20.19)	20.51 (18.17–25.01)	<0.0001
CRP, mg/l, median (IQR)	20.40 (18.00–30.90)	33.28 (18.65–77.50)	101.80 (75.75–121.20)	<0.0001
ALT, U/l, median (IQR)	164.00 (141.00–188.00)	191.00 (125.00–289.00)	286.00 (152.00–383.50)	0.01
AST, U/l, median (IQR)	172.00 (141.00–198.00)	199.50 (148.00–287.00)	266.75 (139.00–388.50)	0.01
γ-GGT, U/l, median (IQR)	488.00 (414.00–564.00)	611.50 (422.00–745.00)	752.00 (590.50–887.50)	<0.0001
DBIL, mg/dl, median (IQR)	48.00 (38.90–55.60)	69.75 (42.60–94.70)	81.80 (71.65–98.05)	<0.0001

AFC, age at first cholangitis; ALT, alanine aminotransferase; AST, aspartate aminotransferase; DBL, direct bilirubin; IQR, interquartile range; KPE, Kasai portoenterostomy; MEPM, Meropenem; T, temperature; WBC, white blood cell; CRP, C-reactive protein; γ-GT, gamma-glutamyl transferase.

Median age was compared using Mann–Whitney test; Age at operation and DBIL were compared using independent samples *t*-test.

**Table 3:** Therapeutic effect of MEPM for mild, moderate and severe cholangitis.

	Mild cholangitis	Moderate cholangitis	Severe cholangitis	*P*
*N*	33	90	28	
DOF (h), median (IQR)	28.00 (24.00–40.00)	36.00 (24.00–48.00)	68.00 (60.00–86.00)	<0.0001
LOS, days, median (IQR)	8.00 (7.00–9.00)	10.00 (8.00–13.00)	15.00 (14.00–16.00)	<0.0001
Age at KPE ≤60 days				
*N*	22	40	4	
DOF (h), median (IQR)	25.0 (24.0–48.0)	42.0 (24.0–64.0)	81.0 (74.5–90.5)	<0.0001
LOS, days, median (IQR)	8.0 (7.0–9.0)	8.0 (7.0–10.0)	13.5 (12.0–14.0)	<0.0001
Age at KPE >60 days				
*N*	11	50	24	
DOF (h), median (IQR)	30.0 (24.0–36.0)	35.5 (25.0–48.0)	68.0 (58.0–86.0)	<0.0001
LOS, days, median (IQR)	8.0 (6.0–9.0)	11.5 (8.0–15.0)	15.0 (14.0–17.0)	<0.0001
	Mild vs. moderate cholangitis	Mild vs. severe cholangitis	Moderate vs. severe cholangitis	
	Mean difference, *P*		
DOF (h)	−9.09, 0.014	−40.52, 0.000	−31.43, 0.000	
LOS, days	−2.68, 0.000	−7.33, 0.000	−4.66, 0.000	
Age at KPE ≤60 days				
DOF (h), median (IQR)	−12.92, <0.0001	−52.27, 0.0001	−39.35, 0.0001	
LOS, days, median (IQR)	−0.65, 0.143	−4.77, 0.0001	−4.13, 0.0001	
Age at KPE >60 days				
DOF (h), median (IQR)	−5.50, 0.342	−37.67, <0.0001	−32.18, 0.0001	
LOS, days, median (IQR)	−3.88, 0.0001	−7.71, 0.0001	−3.83, 0.0001	

DOF, duration of fever; IQR, interquartile range; KPE, Kasai portoenterostomy; LOS, length of hospital stay.

DOF, positive blood culture rates and LOS were compared between the three groups using Kruskal–Wallis test.

The mean difference of the primary and secondary outcomes was compared using One-Way ANOVA.

To reduce the use of carbapenems, antibiotics with lower bacterial coverage, such as CSTS, may provide similar results in the treatment of mild cholangitis. To test this hypothesis, patients with mild cholangitis were randomized to receive either CSTS or MEPM. We found that the DOF of patients who received CSTS was 30.00 (IQR: 24.00–36.00) h, which was not different from that of patients who received MEPM 28.00 (IQR: 24.00–40.00) h (*P*=0.733) (Table 4). This observation confirmed that CSTS was as effective as MEPM and can still be used as a first-line antibiotic for mild cholangitis.

**Table 4:** Primary and secondary outcomes.

	Mild cholangitis			Severe cholangitis		
	CSTS *N*=34	MEPM *N*=33	*P*	MEPM+IVIG *N*=26	MEPM *N*=28	*P*
DOF, h, median (IQR)	30.00 (24.00–36.00)	28.00 (24.00–40.00)	0.73	52.00 (43.00–66.00)	68.00 (60.00–86.00)	0.00
Positive blood culture, *n* (%)	5 (14.7)	3 (9.09)	0.71	6 (23.08)	8 (28.57)	0.76
LOS, days, median (IQR)	7.00 (6.00–9.00)	8.00 (7.00–9.00)	0.35	11.00 (8.00–13.00)	15.00 (14.00–16.00)	<0.0001

DOF, duration of fever; IQR, interquartile range; LOS, length of hospital stay.

DOF and LOS were compared using independent samples *t*-test. The positive blood culture rates were compared *χ* ^2^-test.

To shorten the duration of antibiotic treatment, an effective adjunctive treatment may be needed in patients with severe cholangitis^13^. In this study, patients with severe cholangitis were randomized to receive MEPM with or without IVIG. We found that, patients receiving MEPM+IVIG had significantly shorter DOF than those treated with MEPM alone [52.00 (IQR: 43.00–66.00) h vs. 68.00 (IQR: 60.00–86.00) h, *P*=0.001] (Table 4). The observation suggested that the supplementation of IVIG with MEPM could rapidly improve the anti-infection ability of the infants and reduce the DOF.

### Secondary outcomes

#### Length of hospital stay

In patients with mild to severe cholangitis, LOS was also significantly different among the three groups treated with MEPM [8.00 (IQR: 7.00–9.00) days vs. 10.00 (IQR: 8.00–13.00) days vs. 15.00 (IQR: 14.00–16.00) days, *P*<0.0001] (Table 3). Furthermore, patients with severe cholangitis had the longest LOS, followed by patients with mild and moderate cholangitis. The same results were found in the infants with KPE age greater than 60 days. In the infants with KPE age less than or equal to 60 days, patients with severe cholangitis had the significantly longer LOS than those with mild or moderate cholangitis (Table 3).

In the mild cholangitis group, the LOS of patients who received CSTS was 7.00 (IQR: 6.00–9.00) days, which was not different from that of patients who received MEPM 8.00 (IQR: 7.00–9.00) days (*P*=0.347) (Table 4). In the severe cholangitis group, patients receiving MEPM+IVIG had a shorter LOS than those treated with MEPM alone [11.00 (IQR: 8.00–13.00) days vs. 15.00 (IQR: 14.00–16.00) days, *P*<0.001] (Table 4). For patients with mild cholangitis, CSTS was as effective as MEPM. However, for severe cholangitis, IVIG achieved better results as an add-on treatment to MEPM.

#### Blood culture results

Of the 211 patients, blood culture was positive in 32 patients (15.17%) (Table 5). The positive rates of blood culture were 11.94% in mild cholangitis, 11.11% in moderate cholangitis, and 25.93% in severe cholangitis. The difference in positive blood culture rates between the three groups was significant (*P*=0.038). These observations were consistent with previous studies^7,21^. The most common pathogenic bacteria in moderate and severe cholangitis were Enterococcus faecalis (33.33% of moderate cholangitis and 42.86% of severe cholangitis). However, none of the patients with mild cholangitis were positive for Enterococcus faecalis (Table 5).

**Table 5:** Blood culture results.

Species	Mild cholangitis *N*=67	Moderate cholangitisx *N*=90	Severe cholangitis *N*=54	*P*
*Escherichia coli*	2	1	2	0.70
*Enterobacter aerogenes*	2	2	1	0.91
*Klebsiella pneumoniae*	2	2	1	0.91
*Enterococcus faecalis*	0	3	6	0.01
*Pseudomonas aeruginosa*	1	2	3	0.50
*Enterobacter cloacae*	1	0	1	0.33
Total	8	10	14	0.04

The positive blood culture rates were compared Fisher’s exact test.

#### Secondary outcomes during follow-up

At 7-day follow-up, in the mild cholangitis group, 31 (91.18%) patients treated with CSTS and 31 (90.91%) patients treated with MEPM achieved normal body temperature (*P*>0.05). In the severe cholangitis group, 23 (88.46%) patients treated with MEPM+IVIG and 21 (80.77%) patients treated with MEPM achieved normal body temperature (*P*>0.05) (Table 6).

**Table 6:** Secondary outcomes during follow-up.

	Mild cholangitis			Severe cholangitis		
	CSTS	MEPM	*P*	MEPM+IVIG	MEPM	*P*
7-day follow-up						
*** *** Normal temperature, *n* (%)	31 (91.18)	30 (90.91)	0.97	23 (88.46)	21 (80.77)	0.70
*** *** WBC, ×109/l, median (IQR)	8.27 (6.80–9.98)	7.65 (5.80–8.30)	0.03	10.50 (9.50–11.97)	16.40 (11.50–18.50)	<0.0001
*** *** EUC, XX, median (IQR)	3.83 (2.83–4.90)	3.20 (2.60–3.80)	0.10	5.15 (4.26–7.45)	10.10 (5.60–13.50)	0.00
*** *** CRP, mg/l, median (IQR)	3.41 (1.70–6.00)	5.30 (4.20–8.00)	0.12	16.30 (7.00–21.00)	38.70 (9.70–62.00)	0.00
*** *** ALT, U/L, median (IQR)	109.00 (63.00–188.00)	136.00 (102.00–167.00)	0.78	158.50 (113.00–236.00)	258.00 (144.00–326.00)	0.01
*** *** AST, U/L, median (IQR)	130.50 (87.00–170.00)	130.00 (92.00–170.00)	0.98	167.10 (104.00–233.00)	244.00 (159.00–312.00)	0.01
*** *** γ-GGT, U/L, median (IQR)	350.50 (198.00–554.00)	436.00 (288.00–506.00)	0.60	588.50 (447.00–798.00)	697.00 (525.00–858.00)	0.02
*** *** DBIL, mg/dl, median (IQR)	29.75 (19.50–40.00)	31.00 (23.90–40.00)	0.95	56.80 (37.00–65.00)	66.40 (53.10–89.00)	0.00
1-month follow-up						
*** *** Outcome, *n* (%)						
*** *** NLS	33 (97.06)	31 (93.94)	0.60	24 (92.31)	25 (92.59)	0.97
*** *** LT	0 (0.00)	1 (3.03)		1 (3.85)	1 (3.7)	
*** *** Death	0 (0.00)	0 (0.00)		1 (3.85)	1 (3.7)	
*** *** Lost to follow-up	1 (2.94)	1 (3.03)		0 (0.00)	0 (0.00)	
*** *** Recurrent rate (%)	2 (6.06)	1 (3.23)	0.59	4 (16.67)	10 (40.00)	0.07
*** *** Jaundice clearance rate (%)	27 (81.82)	28 (90.32)	0.32	14 (58.33)	9 (36.00)	0.12
*** *** ALT, U/l, median (IQR)	104.00 (78.00–145.00)	88.00 (65.00–125.00)	0.81	149.50 (88.00–210.50)	196.00 (158.00–251.00)	0.02
*** *** AST, U/l, median (IQR)	106.00 (76.00–164.00)	90.00 (61.00–125.00)	0.18	118.50 (81.50–197.50)	169.00 (156.00–225.00)	0.01
*** *** γ-GGT, U/l, median (IQR)	357.00 (293.00–462.00)	271.00 (113.00–462.00)	0.31	479.00 (352.50–631.50)	654.00 (481.00–847.00)	0.01
*** *** DBIL, mg/dl, median (IQR)	19.40 (15.40–30.90)	19.00 (7.81–32.30)	0.79	45.41 (10.80–72.57)	55.20 (33.60–90.93)	0.09
*** *** 3-month follow-up						
*** *** Outcome, *n* (%)			0.80			0.97
*** *** NLS	29 (85.29)	29 (87.88)		19 (73.08)	19 (67.86)	
*** *** LT	1 (2.94)	1 (3.03)		3 (11.54)	4 (14.29)	
*** *** Death	1 (2.94)	0 (0.00)		2 (7.69)	3 (10.71)	
*** *** Lost to follow-up	3 (8.82)	3 (9.09)		2 (7.69)	2 (7.14)	
*** *** Recurrent rate (%)	5 (17.24)	5 (17.24)	1.00	10 (52.63)	10 (52.63)	1.00
*** *** Jaundice clearance rate (%)	26 (89.66)	27 (93.10)	0.64	8 (42.11)	7 (36.84)	0.74
*** *** ALT, U/l, median (IQR)	73.00 (44.00–89.00)	83.00 (64.00–99.00)	0.39	164.00 (66.00–229.00)	126.00 (78.00–159.00)	0.80
*** *** AST, U/l, median (IQR)	81.60 (41.00–118.00)	76.00 (62.00–99.00)	0.57	123.00 (67.00–189.00)	104.00 (64.00–158.00)	0.91
*** *** γ-GGT, U/l, median (IQR)	298.00 (247.00–341.00)	247.00 (139.00–366.00)	0.72	515.00 (276.00–758.00)	521.00 (347.00–568.00)	0.78
*** *** DBIL, mg/dl, median (IQR)	11.90 (6.60–23.70)	16.00 (3.80–25.50)	0.89	25.40 (8.30–64.00)	35.00 (26.50–55.50)	0.71
*** *** 6-month follow-up						
*** *** Outcome, *n* (%)			0.77			0.97
*** *** NLS	26 (76.47)	27 (81.82)		17 (65.38)	17 (60.71)	
*** *** LT	2 (5.88)	2 (6.06)		4 (15.38)	5 (17.86)	
*** *** Death	1 (2.94)	0 (0.00)		2 (7.69)	3 (10.71)	
*** *** Lost to follow-up	5 (14.71)	4 (12.12)		3 (11.54)	3 (10.71)	
*** *** Recurrent rate (%)	5 (19.23)	8 (29.63)	0.38	13 (76.47)	11 (64.71)	0.45
*** *** Jaundice clearance rate (%)	23 (88.46)	25 (92.59)	0.61	12 (70.59)	10 (58.82)	0.47
*** *** ALT, U/l, median (IQR)	71.60 (45.00–88.00)	79.00 (47.00–95.00)	0.94	116.00 (69.00–189.00)	99.00 (89.00–161.00)	0.54
*** *** AST, U/l, median (IQR)	79.50 (51.00–112.60)	75.00 (49.00–87.00)	0.50	125.00 (83.00–153.00)	119.00 (82.00–135.00)	0.60
*** *** γ-GGT, U/l, median (IQR)	237.20 (143.00–288.00)	217.00 (138.00–339.00)	0.68	509.00 (358.00–595.00)	428.00 (339.00–648.00)	0.80
*** *** DBIL, mg/dl, median (IQR)	7.71 (4.20–19.90)	9.60 (4.40–14.40)	0.74	21.00 (10.50–44.20)	22.00 (11.00–39.10)	0.83

AFC, age at first cholangitis; ALT, alanine aminotransferase; AST, aspartate aminotransferase; CRP, C-reactive protein; CSTS, cefoperazone sodium tazobactam sodium; DBIL, direct bilirubin; IQR, interquartile range; IVIG, intravenous immunoglobulin; LT, liver transplantation; Meropenem, MEPM; NLS, native liver survival; T, temperature; WBC, white blood cell count; γ-GT, gamma-glutamyl transferase.

WBC, EUC, CRP, ALT, AST, γ-GGT, and DBIL were compared using independent samples *t*-test. Outcome, recurrent rate, jaundice clearance rate was compared using *χ* ^2^ test.

In the mild cholangitis group, patients treated with MEPM had lower WBC than those treated with CSTS [7.65 (IQR: 5.80–8.30) vs. 8.27 (IQR: 6.80–9.98), *P*=0.032]. There was no significant difference in EUC, CRP, ALT, AST, γ-GGT, and DBIL between CSTS and MEPM groups (all *P*>0.05) (Table 6). In addition, in patients with severe cholangitis, MEPM+IVIG significantly decreased WBC, EUC, CRP, ALT, AST, γ-GGT, and DBIL compared to MEPM (all *P*<0.05) (Table 6).

At 1-month follow-up, the jaundice clearance rate in the mild cholangitis group was not different between those treated with CSTS and MEPM (CSTS, 81.82% vs. MEPM, 90.32%). There was no significant difference in NLS, relapse rate, ALT, AST, γ-GGT, and DBIL between the two groups (all *P*>0.05) (Table 6). Furthermore, in the severe cholangitis group, patients treated with MEPM+IVIG had lower ALT [149.50 (IQR: 88.00–210.50) vs. 196.00 (IQR: 158.00–251.00), *P*=0.016], AST [118.50 (IQR: 81.50–197.50) vs. 169.00 (IQR: 156.00–225.00), *P*=0.009] and γ-GGT [479.00 (IQR: 352.50–631.50) vs. 654.00 (IQR: 481.00–847.00), *P*=0.014] than those treated with MEPM (Table 6). However, there was no significant difference in NLS, recurrence rate, jaundice clearance rate, and DBIL between the MEPM+IVIG group and the MEPM group (all *P*>0.05).

At 3 and 6 months of follow-up, in the mild cholangitis group, there was no significant difference of NLS, recurrence rate, jaundice clearance rate, ALT, AST, γ-GGT, and DBIL between the patients treated with CSTS or MEPM (all *P*>0.05) (Table 6). Similar results were observed in the severe cholangitis group. No significant differences in NLS, recurrence rate, jaundice clearance rate, ALT, AST, γ-GGT, and DBIL were found between the MEPM+IVIG group and the MEPM group (all *P*>0.05) (Table 6).

At 7-day follow-up, no recurrent cholangitis was observed in the study population. At 1-month follow-up, the incidence of recurrent cholangitis was significantly lower in the mild cholangitis group than in the severe cholangitis group (4.69 vs. 28.57%, *P*=0.0009). Similar results were found at 3 months (mild, 17.24% vs. severe, 52.63%, *P*=0.0006) and 6 months (mild, 24.53% vs. severe, 70.59%, <0.0001) of follow-up.

These results indicated that CSTS and MEPM treatment had similar outcomes in mild cholangitis at 6 months of follow-up, supporting CSTS as a first-line antibiotic for mild post-KPE cholangitis. Furthermore, in severe cholangitis, MEPM+IVIG improved liver function at 7 days and 1-month after enrollment. The results suggest that IVIG is an effective adjunctive treatment for severe post-KPE cholangitis to improve short-term clinical outcomes.

## Discussion

This study developed a severity grading system to guide empiric antibacterial therapy for post-KPE cholangitis. We found that CSTS was as effective as MEPM for mild cholangitis. MEPM in combination with IVIG may be necessary in patients with severe cholangitis. We believe that severity-guided antibiotic therapy could reduce broad-spectrum antibiotic treatment for mild cholangitis and improve clinical outcomes in severe cholangitis.

Treatment of post-KPE cholangitis is challenging, because the microbial causes are often unknown^4,7,8^. In this study, blood cultures were positive in 15.17% of our cohorts. Early empiric antibiotic treatment should be directed against all likely causative bacteria^7,21^. It is reported that the most commonly isolated bacteria in cholangitis after KPE were Escherichia coli, Klebsiella pneumoniae, Pseudomonas aeruginosa, Enterobacter cloacae, and Enterococcus faecium^1,12^. In this study, Enterococcus faecium (9, 28.1%) was the most frequently isolated bacteria, followed by Pseudomonas aeruginosa, Escherichia coli, Enterobacter aerogenes, Klebsiella pneumoniae, and Enterobacter cloacae.

Cefoperazone was found to be superior in the treatment of acute cholangitis, with a primary response rate of 88.9%^10^. After years of use as a first-line empiric antibiotic, the efficacy of cefoperazone had decreased to 75%^10,11^. In recent years, MEPM has been shown to be effective for post-KPE cholangitis because its broad-spectrum and good biliary concentration^10,22^. Recent studies have recommended MEPM as a suitable candidate for cefoperazone, especially in nonresponders or infants who have previously received piperacillin-tazobactam or cefoperazone^11,12,23^. However, whether the use of MEPM improves clinical outcomes compared with the use of cefoperazone in mild post-KPE cholangitis remains controversial. In addition, more than half of the patients eventually required a change in antibiotics because some severe cases do not respond to initial medical treatment^10^.

For these reasons, it is ideal to select empirical antibiotics according to the severity of the clinical presentation. We developed a simple severity grading system to classify post-KPE cholangitis into three categories: mild, moderate, and severe. Moderate cholangitis was treated with MEPM because studies consistently showed that MEPM was an effective empirical antibiotics for post-KPE cholangitis^10,22,24^. We randomized the mild cholangitis patients into the CSTS or MEPM treatment group. We found that the CSTS produced similar clinical outcomes in mild cholangitis as compared with the MEPM. These observations support CSTS rather than MEPM as a first-line antibiotic for mild post-KPE cholangitis.

Previous studies have shown that IVIG supplementation in severe infections rapidly improved immune function and anti-infective capacity in children^13,17,19^. In addition, high-dose IgG resulted in decreased bilirubin, bile duct inflammation, and increased extrahepatic bile duct patency^11^. We previously reported that IVIG was an effective adjunctive treatment for intractable post-KPE cholangitis^13^. In this study, we found that, in severe cholangitis, MEPM+IVIG significantly improved clinical outcomes compared with MEPM alone, as those patients had shorter DOF and LOS. In addition, in the severe cholangitis group, IVIG significantly improved liver function at 7 days and 1-month, suggesting that an antibody-mediated mechanism is involved in post-KPE cholangitis. Previous studies have shown that abnormal immunophenotypes are associated with the development of cholangitis and the long-term outcome of BA^25,26^. IVIG has a variety of effects on inhibiting T-cell activation, antibody and cytokine production, dendritic cell maturation, natural killer cell trafficking, and the expansion and activation of anti-inflammatory regulatory T cells^27,28^. It is possible that IVIG could alleviate post-KPE cholangitis through its direct effects on altering the immunophenotype. A future clinical trial would provide a more definitive conclusion on the efficacy and feasibility of IVIG in the early phase of post-KPE cholangitis.

To our knowledge, the severity grading system developed in the present study is the first feasible tool in the management of post-KPE cholangitis. However, our study is an open-label prospective study with a relatively small sample size. We only analyzed the outcome of the following 6 months. Long-term follow-up results should be evaluated in a future study.

## Conclusions

In conclusion, empiric antibiotic treatment should be based on the results of the severity grading system. For mild cholangitis, cefoperazone is the first-line antibiotic. MEPM is the optimal antibiotic treatment for moderate cholangitis. MEPM plus immunoglobulin treatment could be used for those with severe cholangitis.

## Ethical approval

Ethical approval for this study (Ethical Committee S124) was provided by the Ethical Committee of Tongji Medical College of Huazhong University of Science and Technology on 10 June 2020.

## Consent

Written informed consent was obtained from the patient for publication of this case report and accompanying images. A copy of the written consent is available for review by the Editor-in-Chief of this journal on request.

## Sources of funding

This research was funded by the National Natural Science Foundation of China (81873541, Jiexiong Feng) and Tongji Hospital Clinical Research Flagship Program (2019YBKY026, Jiexiong Feng).

## Author contribution

J.F. and H.R.: conceptualized and designed the main framework of this study; P.W. and H.-Y. Z.: data collection, analysis and interpretation, drafting of the manuscript; J.Y., T.Z., X.W., B.Y., X.S., B.W., W.T., J.T., X.L., J.Z., Y.L., Y.L., Z.L., T.F., D.W., W.L., and H.Y.: provided study materials or patients. All authors agree that they have been involved in the review and critical revision of the manuscript for important intellectual content, and have read and approved the final draft for submission. All authors agree to take responsibility for the content of this study.

## Conflicts of interest disclosure

The authors declare that they have no conflicts of interest.

## Research registration unique identifying number (UIN)

1. Name of the registry: Clinical Trials.gov.
2. Unique identifying number or registration ID: NCT04370145.
3. Hyperlink to your specific registration (must be publicly accessible and will be checked): https://clinicaltrials.gov/ct2/show/NCT04370145.

## Guarantor

Jiexiong Feng and Hongxia Ren.

## Data availability statement

The authors confirm that the data supporting the results of this study are available in the article and its supplementary materials. In addition, any additional data from this study are available upon request from the corresponding author.

## Provenance and peer review

The paper was not invited.
